# Supplementary material for: Are carbon emissions trading and green financial instruments synergistic? -Comprehensive quantitative research based on content analysis
Source: PLoS One. 2024 Mar 7;19(3):e0298601. doi: 10.1371/journal.pone.0298601 (PMC10919601; doi:10.1371/journal.pone.0298601)
Supplement: S1 Data — (ZIP) [file pone.0298601.s001.zip › Data/地级市Z_F工作报告.zip!/┴╔─■/╜⌡╓▌╩╨/└┤╫╘╣1⁄2╓┌║┼í╛┬φ┐╦╩2╛▌═°í┐_2009.docx]

各位代表：

现在，我代表市政府向大会报告工作，请予审议，并请市政协各位委员提出意见和建议。

一、2008年工作回顾

2008年，是不同寻常的一年。在省委、省政府和市委的正确领导下，我们紧紧依靠和团结全市人民，以科学发展观为统领，抢抓机遇，攻坚克难，较好地完成了市第十四届人民代表大会第一次会议确定的各项任务，经济社会发展取得新的重大成就。

初步核算，全市地区生产总值实现690.4亿元，增长15%；万元GDP综合能耗降低5.3%；全社会固定资产投资完成248.7亿元，增长48.4%；地方财政一般预算收入完成35.1亿元，增长35%；社会消费品零售总额实现228.1亿元，增长23.1%；实际利用外资3.1亿美元，增长114.2%；实际利用域外资金112亿元，增长104%；城镇居民人均可支配收入达到13962.5元，增长16.3%；农民人均纯收入达到6089.4元，增长20.6%。

(一)锦州湾开发实现新突破

积极抢抓省“五点一线”战略机遇，锦州湾开发全面快速推进。10平方公里的锦州港、103平方公里的娘娘宫临港产业区和125平方公里的凌海大有临海经济产业区成功纳入省沿海经济带建设重点支持区域，以西海工业园区、白马综合工业园区、娘娘宫临港产业区和白沙湾行政生活区为主体的滨海新区发展格局初步形成。

锦州港建设取得重大进展。高天铁路增能扩建工程全部完工，港口实现国铁直进直出。西部海域开发进展顺利，中电投、华润电力锦州港煤炭专业码头等项目正式签约。锦州港向大连港非公开发行股票24600万股，筹集港建资金20亿元。锦州港作为蒙东及蒙古国出海口工作实现新突破，新锦赤铁路开工建设。港口吞吐量完成4723万吨，集装箱运输完成65万标箱，分别增长34.4%和44.4%。

西海、白马工业园区建设快速推进。中海街、沈阳路等9条街路建成通车。热电管网、一次变电站、标准厂房等基础设施全面开工建设。光伏和汽车零部件产业园配套设施基本完成，100兆瓦太阳能电池、砂浆回收等10个项目建成投产，2000吨单晶硅片、启明汽车电子等18个项目开工建设。华润电厂、国家石油战略储备基地等一批重大项目顺利推进。

白沙湾建设全面展开。供水、供电、供暖、燃气等基础配套设施日趋完善，学校、医院等服务设施相继开工建设，房地产开工面积达到88万平方米。滨海路海滨浴场至三角山段建成通车，娘娘宫段建设全面启动，锦州湾大桥完成主体工程。

娘娘宫海域开发进展顺利。娘娘宫临港产业区概念性规划和船舶工业园发展总体规划编制完成。修造船园区围堰及海防堤工程全面开工，2平方公里起步区回填工程如期完成，3家企业签订入园协议。

(二)工业经济持续快速增长

全年共安排固定资产投资500万元以上工业项目633个，其中373个项目建成投产。工业固定资产投资达到139.9亿元，增长71.6%。规模以上工业增加值完成216.3亿元，增长16%。全市亿元以上企业达到125户。

工业结构调整步伐加快。光伏、汽车零部件、精细化工等新兴支柱产业快速壮大，新世纪石英玻璃1000吨低成本多晶硅、配天汽车电子、惠发天合氟碳醇等一批重点项目建设进展顺利，部分项目竣工投产。石化、铁合金、农产品加工等传统优势产业实力进一步增强，锦州石化130万吨加氢裂化、中信铁合金中低碳锰铁扩能改造等项目完成大部分投资，大成食品20万吨淀粉糖、汇源集团18万吨果汁等项目全部投产。女儿河一次变、二次变投入使用，大唐热电、恒亚内燃机、国电凌海风电等一批项目顺利推进。

企业自主创新能力进一步提高。创建省级科技创新示范企业5家。成立省级工程技术研究中心9个、企业技术中心14个。58个项目列入国家、省级科技计划。完成专利申请440件。高新技术产品增加值增长30%。

节能减排工作扎实推进。关停排污大户2家，关闭污染严重企业13家。创建国家级环保模范城10项重点工程完成7项。全市9座污水处理厂建设有序推进。全年减排化学需氧量35000吨、二氧化硫4000吨。城区空气质量优良率达到94.3%。

国企改革实现新的突破。出台了《锦州市推进国有企业改革总体实施方案》。重型、女纸等10户企业改革稳步推进，退出国有资本2.9亿元。辽晶、矿山机器厂等5户企业完成政策性破产。成功回购金融类债权4.8亿元，释放抵押资产价值2亿元。

(三)县域经济发展势头强劲

县域工业迅猛发展。按照“双增升位”总体目标要求，以发展县域工业园区为主攻方向，加大基础设施建设力度，强化招商引资工作，加快主导产业培育，强力推进县域经济发展。庞河、沟帮子、大有、七里河四个工业先导区完成起步区规划，基础设施建设投资累计达到2.5亿元。四县(市)共安排固定资产投资500万元以上项目400个；规模以上工业增加值完成92.2亿元，增长25.1%；财政一般预算收入完成11.3亿元，增长55%，创历史最好水平。

农业综合生产能力显著增强。预计农林牧渔业增加值实现124亿元，粮食总产量达到21.05亿公斤，肉蛋奶总产量达到100万吨。新上和扩改农业产业化项目50个。新增千亩以上农业科技示范小区70个。订单农业达到240万亩。设施农业达到135.4万亩，居全省第一位。新建省级标准化畜牧养殖小区387个，重大动物疫情连续3年为零。渔业资源修复和健康养殖取得新发展。新组建农民专业合作社213家。增加有效降雨3.2亿立方米。农业综合机械化水平达到61%。社会口径转移农村劳动力5.5万人。

农村生产生活环境不断改善。新安排土地开发整理项目16个，耕地总量连续10年正增长。增加水浇地13.8万亩，改善治涝15万亩，治理水土流失12万亩。造林35万亩，封山育林10.2万亩。建设“绿化新村”124个。集体林权改革完成97.5%。完成整村推进扶贫项目47个，3.9万农村低收入人口实现稳定脱贫。建设饮水工程86处，解决了10.8万人的饮水安全问题。新增农村清洁能源用户1.1万户。新建乡镇客运站11座，完成村通油路328公里。

(四)服务业发展水平稳步提升

服务业发展摆上重要位置。将现代服务业列为全市重点发展的支柱产业，成立了市服务业发展领导小组，出台了《关于加快发展服务业若干政策措施的实施意见》。

主要商业街区建设全面启动。确立了以中央大街和上海路为两轴、古塔区和凌河区为两翼的中心商业街区改造计划，辽西国际商城等4个大项目、12个子项目的招商引资工作全面展开。

重点项目建设快速推进。锦州港粮食物流一期投入试运营，渤海物流和恒大物流二期开工建设。辽西小商品市场东扩、义县义乌商品城等项目顺利竣工，锦州国际酒店、万博国际会展中心等项目完成主体工程。新建、改建连锁农家店1100家。北镇窟窿台蔬菜批发市场通过兼并收购成为东北最大的农产品批发市场。

旅游业发展逐渐升温。奉国寺和大芦花申报国家4A级景区工作通过省初检。红色旅游、蓝色旅游、金色旅游等品牌日趋成熟。旅游总收入增长62%。

(五)对外开放迈出新步伐

招商引资工作势头良好。理顺管理体制，强化队伍建设，拓展招商领域，创新工作方式，成功举办了香港、“长三角”、“珠三角”等六次高密度、大规模招商活动，同深圳、温州、台州等地客商建立了稳定广泛的联系，香港ITAT服装交易中心、90万吨焦炭等一批商业地产和工业项目落户锦州。县域招商活动日趋活跃，围绕工业园区建设和主导产业培育开展的集群式招商收效明显。狠抓现有企业增资扩能，增资项目到位外资1.9亿美元。

外贸出口工作在困境中推进。加大对机电、光伏等高技术含量、高附加值产品出口的政策扶持力度，出口产品结构不断优化。克服国家出口政策调整等不利因素，促成了中石油国际事业公司、宏丰印染等企业加工贸易出口。出口创汇预计完成7.2亿美元，其中机电产品出口完成1.78亿美元，增长38.1%。

(六)城市环境进一步优化

城市规划水平明显提升。立足于锦州未来发展和城市整体形象的改观，对新老城区建设与改造进行了更高标准的规划。结合老城区改造和城市南扩，确定了未来三年城市建设十大工程。

重点城建工程顺利实施。云飞南街女儿河斜拉桥建成通车。小凌河福州街人行桥、东湖森林公园二期工程如期完工。新建、大修、拓宽宜昌路等主次干道16条，中央大街、市府路等6条主要街路完成路灯改造。新增城市绿地3.9万平方米。二热电三期扩建工程建成投入运行。凌南地区燃气管网环网工程顺利竣工。接收改造二次供水系统70座。锦州公路客运中心站投入运营。5.4公里锦汤快速通道建成通车。

城市管理工作进一步加强。城市管理相对集中行政处罚权全面实施。市容整治专项行动取得明显成效，店外烧烤、马路市场和站前脏、乱、差等问题得到较好解决。100条街路实施网格化管理，实现了与“门前三包”工作的有效对接。

(七)财税金融工作平稳运行

财税工作扎实有效。充分利用财政贴息和税收政策，积极争取上级资金，保证了重点项目、重点企业和县域工业园区建设的顺利进行。加大重点税源企业、零散税收和非税收入的征管力度，严厉打击偷逃骗税等违法行为，税费征管秩序进一步规范。严格控制支出，妥善调度资金，支出结构不断优化。深化国库集中支付制度改革，简化政府采购手续，加强国有资产监管，财政改革迈出新的步伐。

金融服务能力不断增强。加强金融生态环境建设，有效防范和化解金融风险，全市银行信贷资金良性运转，金融机构本外币存贷款余额分别比年初增长19.5%和19.1%。锦州银行成为全国首家跨省经营的地级城市商业银行。阳光能源公司在香港成功上市。全市保险主体发展到22家。

（八）各项社会事业协调发展

科技、人才、教育工作稳步推进。成功举办了中国(锦州)北方农业新品种、新技术展销会。培训农民科技经纪人237人，培养农村科技人才105人。引进外国专家220人次。“教育质量年”活动扎实有效。凌海“双高普九”和黑山、太和“提高普九”通过省评估验收。新建农村九年一贯制学校13所，改造中小学危房2.7万平方米。

文化、体育、广播电视工作成果喜人。成功举办了古塔庙会、古玩文化节、国际民间文化节和纪念锦州解放60周年等大型节庆活动。满族民间刺绣等3个项目入选第二批国家级非物质文化遗产名录。北京奥运会上我市运动健儿喜获两金两银佳绩。十大电视新闻人物评选等系列活动影响广泛。率先在全省完成广播电视村村通工程。

医疗卫生、计划生育工作成效明显。全面完成乡镇卫生院建设，实现了村村有甲级卫生室目标，新农合农村常住人口参合率达到100%。建立了城市居民健康档案，社区卫生服务功能不断提升，覆盖率达到100%。计划生育家庭奖励扶助政策进一步落实，人口出生率和自然增长率分别为7.86‰和1.27‰。

精神文明建设等工作全面推进。市民素质提升、和谐社区创建、行业服务创优等创建活动卓有成效。第八届社区居民委员会换届选举工作圆满完成。双拥工作成果进一步巩固。“五五”普法和法律“六进”等宣传活动扎实深入。支援四川震区救灾和重建工作空前踊跃。统计、档案、人防、地震、慈善、残联、社会福利、民族宗教、外事侨务等工作取得新的成绩。

（九）民生工程取得明显成效

就业再就业工作成效显著。开发公益性岗位，建立就业安置基地，拓宽就业渠道，扩大就业容量，实名制安置就业、普惠制就业培训和组织劳务输出分别完成年度计划的231.2%、178.9%和178.6%。全年基本保持零就业家庭动态为零。城镇登记失业率控制在3.8%以内。

社会保障体系日趋完善。城镇职工参加基本养老、医疗、失业、工伤、生育保险人数分别达到57、60.4、39.4、24.8、14.3万人。城市和农村低保对象保障面分别达到6.6%和4.2%。年归集住房公积金6亿元。完成廉租房建设2.2万平方米。兰花里1700余户居民迁入新居，兴业里、工学里等棚户区改造完成主体工程。

平安稳定工作效果明显。建立和完善信访工作长效机制，市县两级信访部门受理群众来信来访量下降16%。突出做好奥运安保工作，建立反恐防暴应急机制，保持严打高压态势，群众安全感综合排名连续两年全省第一。严格落实安全生产目标责任制，深入开展安全生产专项整治，全年未发生较大安全生产事故，亿元GDP生产安全事故死亡率低于省定指标。

（十）政府自身建设不断加强

依法行政能力进一步增强。自觉接受人大法律监督、政协民主监督和群众社会监督，及时向人大及其常委会报告工作，认真与政协民主协商重要事项。440件代表建议和政协提案办结率、见面率、满意率分别达到100%、100%和98.9%。

软环境建设进一步改善。切实加大行政监察和审计监督力度，成立了软环境建设办公室，出台了《服务承诺制》、《首问责任制》、《限时办结制》、《行政处罚自由裁量权暂行规定》等一系列规章制度，行政行为进一步规范。第四次行政审批制度改革削减行政审批事项77项，行政效率明显提高。

公共服务能力进一步提升。积极推进应急预案体系和信息平台建设，应急管理能力不断增强。大力推行政务公开，有效保障了人民群众知情权、诉求权和监督权。加强机关纪律作风建设，强化廉洁自律教育，政府行政效能和公共服务能力全面提升。

各位代表，过去一年经济社会发展的成绩来之不易，这是省委、省政府和市委正确领导的结果，是全市人民团结奋斗、开拓进取的结果，也得益于历届市委、市政府打下的良好工作基础。在此，我代表市政府，向在各个领域和岗位上辛勤劳动、做出贡献的全市人民表示崇高的敬意！向给予政府工作大力支持的人大代表、政协委员、各民主党派、工商联和各人民团体，向关心和支持政府工作的离退休老同志，向中省直各部门、各单位，向驻锦部队和武警官兵，向所有关心支持锦州发展的海内外朋友，表示衷心的感谢！

回顾过去一年的工作，我们也清醒地看到，全市经济社会发展还存在一些不容回避的困难和问题。主要表现在：工业结构不尽合理，具有规模优势、品牌优势、科技竞争优势的企业还不是很多；县域经济外向度较低，区域间发展还不平衡；外贸出口形势严峻，不利于进一步扩大出口的因素日趋增多；财政保障能力有限，工资、社保等刚性支出与可支配财力之间的矛盾仍较突出；城市基础配套设施建设尚需加强，管理水平有待进一步提高；政府部门服务发展的意识有待加强，软环境建设还存在一些不容忽视的问题，等等。所有这些困难和问题，都需要我们在今后的工作中认真加以解决。

过去的一年，我们在工作实践中也进一步深化了对锦州经济社会发展规律的认识，主要体会是：

第一，必须坚持科学发展思路。科学的思路是发展的重要前提。市委提出的“一纲五线”发展战略契合科学发展观要求，符合锦州发展实际，必须一以贯之地执行，毫不懈怠地为之奋斗。

第二，必须深化改革扩大开放。改革开放是发展的必然选择。只有不断解放思想，以更大的力度推进改革，在更广的领域扩大开放，才能充分调动各方面的积极性，为经济社会发展注入新的生机与活力。

第三，必须更加关注改善民生。改善民生是发展的根本目的。只有始终把人民群众的利益放在首位，真正实现好、维护好、发展好最广大人民的根本利益，让发展成果更多惠及人民群众，才能无愧党的信任和人民的支持。

第四，必须维护社会和谐稳定。社会和谐稳定是发展的坚实基础。只有正确处理改革发展稳定的关系，认真解决群众诉求，积极化解社会矛盾，切实维护公平正义，才能开创和谐稳定的社会局面，确保经济社会又好又快发展。

第五，必须抓实抓好作风建设。作风建设是发展的有力保障。只有不断加强作风建设，大力弘扬“勇于牺牲、敢为人先”的锦州精神，才能形成聚精会神搞建设、一心一意谋发展的强大合力，把各项事业不断推向前进。

二、2009年工作安排

各位代表，今年是新中国成立60周年，也是我们加速辽西沿海经济区中心城市建设的关键之年。我们既面临着国际金融危机带来的复杂多变外部环境的严峻挑战，也面临着国家进一步扩大内需、辽宁沿海经济带建设即将上升为国家战略，以及国内外发达地区产业转移等千载难逢的机遇。

新的一年里，我们一定要全面贯彻党的十七大、十七届三中全会和中央、全省经济工作会议精神，深入贯彻落实科学发展观，按照市委十届六次全会的部署，坚定不移地实施“一纲五线”发展战略，加大基础设施建设力度，加快经济结构调整和发展方式转变，推进改革开放和科技创新，加强节能减排和环境保护，更加重视改善民生和社会建设，千方百计实现“四个确保”，加速推进辽西沿海经济区中心城市建设。

2009年经济社会发展的主要预期目标是：地区生产总值增长15%以上，万元GDP综合能耗和主要污染物排放量均降低5%，全社会固定资产投资增长40%，地方财政一般预算收入增长15%，社会消费品零售总额增长15%，实际利用外资和域外资金分别增长10%和40%，城镇居民人均可支配收入和农民人均纯收入分别增长10%和12%，城镇登记失业率控制在5%以内。

围绕上述目标要求，重点做好以下十个方面工作：

（一）抢抓扩大内需机遇，加快基础设施建设

用足用好中央和省关于扩大内需的相关政策，全力以赴抓好重大基础设施项目建设。全年安排500万元以上基础设施建设项目69个，投资总额295亿元。

加快交通基础设施建设。抓紧做好锦州湾国际机场、城市轻轨、渤海二道等重大项目的前期准备工作，力争年内开工建设。加快滨海公路建设，完成三角山至北小柳段建设工程。进一步完善南站地区交通路网建设。建成乡镇客运站10座。新建村通油路106.2公里，实现所有行政村全部通油路。积极推进中电投、华润电力、大唐国际锦州港煤炭专业码头项目。

加快能源基础设施建设。推进义县生物发电、风力发电项目建设，力促黑山生物发电、北镇稻壳发电和凌海、黑山风电等项目建成投入运营。加快城乡电网改造。抓紧做好大唐热电二期、华润电力2台100万千瓦燃煤机组工程前期工作。

加快农业基础设施建设。积极争取国家和省政策支持，确保锦凌水库3月底前开工建设。加快农村饮水安全工程建设，解决10万人饮水安全问题。新增设施农业小区1600个。建设标准化畜禽养殖小区337个、水产健康示范区26个。抓好44.8公里滨海公路绿化工程。推进农村新型能源建设，建成户用沼气池1.2万个。

积极推进其它领域基础设施建设。加大开放力度，争取上级支持，切实推进工业、服务业、环境设施和文化教育、医疗卫生等各项社会事业的基础设施建设。

（二）加快锦州湾开发开放，推进滨海新区建设

抢抓沿海经济带发展战略机遇，强力推进锦州港、西海工业园、白沙湾行政生活区和娘娘宫海域“四大板块”建设，努力实现锦州湾开发的新突破。

加速推进锦州港建设。加大进港航道浚深、西部海域开发等基础设施建设力度，争取209、210集装箱泊位开工建设。全面推进锦州港作为蒙东及蒙古国物流出海口建设。进一步密切与大连港的合作，力争在航线开辟、海洋资源综合利用等方面取得实质性进展。大力开拓腹地市场，力争港口吞吐量和集装箱运输分别达到5000万吨和75万标箱。

加速推进滨海新区建设。进一步完善西海工业园、白马综合工业园标准厂房等基础设施建设，切实增强园区的项目承载能力。加快恒山路、富海街等“三横五纵”路网建设，推进行政中心、创业中心、文化公园等重点工程，尽快形成白沙湾中心区主体框架。加大娘娘宫临港产业区基础设施建设力度，全力推进船舶修造园起步区和30万吨船坞建设进程，力争引进5-8个修造船项目。加快推进对苯二甲酸、深圳大中华、同洲电子等一批重大在谈项目。

加速推进大有临海经济产业区建设。加大招商引资力度，主动承接临港产业转移，推进塑料、能源动力、金属冶炼加工等产业发展，加快形成沿海特色产业集群。

（三）着力调整产业结构，加快发展方式转变

加快提升自主创新能力。坚持以自主创新推动发展方式转变和产业结构优化升级。加大对光伏、信息技术、生物制药等高科技产业的扶持力度，加速科技成果转化运用，力争在重大技术装备、自主知识产权和新产品、新技术开发上取得新突破。加强科技人才队伍建设，合理配置人才资源。深入实施名牌战略，争取有2-3个产品获评全国名牌产品。

发展壮大新兴支柱产业。大力推进光伏产业“一园三基地”建设，扩大多晶硅、单晶硅及硅片、太阳能电池及组件等光伏产业产能，进一步拉长产业链。加快推进汽车零部件产业园二期工程建设，积极引进规模大、技术高、产业关联度强的项目，尽快形成产业集群。加快石化精细化工园区、义县氟化工园区和中信铁合金钛白粉生产基地建设，不断扩大精细化工产业规模。积极促进华昌光伏太阳能电池、中信铁合金中低碳锰铁扩能改造、惠发天合氟碳醇等一批续建和新开工项目竣工投产，加快推进金纸100万吨制浆造纸、万得电动汽车、佳兴鸿泰200万吨沥青等一批项目建设进程，抓紧做好百万吨乙烯、石化公司10万吨异丙醇、大成食品50万吨多元醇等一批项目的前期准备工作。全年共安排重点工业项目102个，确保规模以上工业增加值增长15%，工业固定资产投资增长20%以上。

全面推动服务业发展。按照改造提升传统服务业、大力发展现代服务业、加快发展新兴服务业的总体思路，加快推进老城区中心商业街区建设，大力发展物流、金融、证券、会展、咨询等新兴服务业，进一步完善城乡市场流通和农副产品市场体系。力争辽西国际商城等4个大项目、12个子项目招商引资工作实现突破性进展，锦州国际酒店、万博国际会展中心等一批在建项目如期竣工。新建改造“万村千乡”连锁农家店220个，社区商业网点230个。采取有效措施，活跃城乡市场，扩大内需，拉动消费。全面提高房地产业发展水平，完成400万平方米建设任务。整合旅游资源，加快项目开发，强化宣传促销，加强区域联动，旅游总收入增长18%。

加强资源节约和生态环境保护。严格执行能耗和环保标准，加强重点耗能企业能源监察审计，深入搞好企业节能目标责任评价考核。继续开展违法排污专项整治活动，严厉打击环境违法行为。加快推进国家环保模范城创建工作，确保9座污水处理厂建成投入使用，全年空气质量达到国家二级标准天数不少于317天。

（四）加快县域经济发展，扎实推进新农村建设

高标准建设县域工业园区。坚定“双增升位”目标不动摇，加快推进县域工业化、城镇化和现代化进程。按照城乡统筹规划、产业合理布局的总体要求，进一步加快庞河、沟帮子、大有、七里河等工业先导区基础设施建设。结合县域产业发展和工业园区建设实际，每个县(市)集中培育一个工业主导产业、一个农业产业化主导产业，努力实现统筹发展、同步推进。

切实提高农业综合生产能力。实现粮食总产量42亿斤。全力推进国家大型商品粮优质稻米生产基地建设，实施玉米、水稻、蔬菜、水果、花生等五大产业提升行动。新建和完善千亩以上农业科技示范区100个。引进推广农业新品种、新技术100项。规模以上龙头企业达到250家。新增农民专业合作社50家。建设农业机械化示范乡镇30个，农业机械化水平达到63%。完成“阳光工程”技能培训9800人，新增转移农村劳动力5万人。

进一步改善农村生产生活环境。严格节约集约用地，优先保障重点区域、重点项目和民生用地。加大基本农田保护和土地开发整理力度，确保耕地动态占补平衡。新增水浇地10万亩，改善治涝10万亩，治理水土流失10.5万亩。启动南山绿化三期和锦凌水库水源涵养林建设工程，造林30万亩、封山育林10万亩，建设“绿化新村”100个。全力做好重大动物疫情防控工作，确保畜禽及产品安全。加强气象防灾减灾能力建设，实现气象防灾减灾信息进村屯。实施整村推进扶贫项目50个，力争2.5万贫困人口实现稳定脱贫。推进新农村村企共建工作，新建村级信息站100个。

（五）深化改革扩大开放，开创开放新的局面

加大改革攻坚力度。以35户市属工业企业改革为重点，加大盘活资产、实施破产等改制工作力度，力争有20户企业完成改制任务。妥善处理金纸、铁合金、重型等企业改革遗留问题。积极推进燃气总公司等公用事业改革，全面完成林权制度改革。

加大招商引资力度。积极抢抓国内外产业转移的难得机遇，进一步加强招商队伍建设，制定出台优惠政策，精心编制包装项目，继续做好“长三角”、“珠三角”等重点区域及韩国、日本、新加坡、香港、台湾等重点国家和地区的招商引资活动。特别是要以工业园区建设为平台，突出抓好主题概念招商、集群式招商、以商招商和行业招商，力求在主导产业数量和质量上有新的突破。

加大出口创汇力度。重点抓好机电、光伏等高技术含量、高附加值产品的出口，不断优化出口商品结构。引导企业进行产品更新、技术升级和节能环保，鼓励无权企业申请进出口经营权，加快培育新的出口增长点。继续改善口岸通关环境，提高通关效率。推进口岸综合查验设施建设，实现口岸查验“一站式”办公、“一条龙”服务。

（六）加强规划建设管理，打造滨海锦州形象

加快推进城市南扩。启动凌南燃气储罐、“九街五路”绿化和南站欧洲风情小镇等工程建设。抓紧推进滨海新区城市规划论证工作，致力形成滨海新区“一港、两核、双翼、五廊”的总体布局。

大力实施老城区改造。开工建设云飞街小凌河大桥、广州街公铁立交桥。启动实施中环西路三期续建、单洞扩孔、站前广场和公交西客站改造工程。完成东湖森林公园三期改造工程。新建、改造北京路、解放路等9条主次干道。积极筹建城市生活垃圾污泥焚烧发电厂。确保大唐供热管线工程投入运行。全面完成社会自管二次泵站接收改造任务。

进一步加强城市管理。全面推行相对集中行政处罚权工作，逐步建立健全城市管理长效机制。强化城市规划监督，严格市容和环境卫生管理，切实解决群众反映强烈的马路市场扰民、机动车侵占人行步道等城市顽疾。继续强化小区物业管理与整治，进一步改善人居环境。

（七）强化财税金融工作，提高管理服务水平

切实增强财税增收保障能力。加强税收征管，强化税源监控，整顿税收秩序，努力实现财政收入与可支配财力的协调增长。充分利用国家扩大内需政策，发挥财政贴息等“四两拨千斤”的作用，支持支柱产业、骨干企业、县域工业园区和民营经济发展。加强机关事业单位资产管理，提高政府筹资能力，拓宽城市基础设施建设融资渠道。继续推进部门预算、国库集中收付、政府采购和非税收支管理改革。强化财政预算管理，优化财政支出结构，确保政权运转、社会保障、民生工程等重点支出。

切实提高金融保险服务水平。大力开展小额贷款公司承贷工作，着力解决中小企业融资难、贷款难等问题。每个县(市)区至少要建立1家中小企业融资担保公司，进一步健全融资担保体系，搭建银企对接平台。切实做好奥鸿、新世纪石英玻璃、新华龙、电力金具、天合精细化工等拟上市企业包装工作，争取有2-3户企业成功上市。加快推进保险业诚信服务和保险市场的健康有序发展。

（八）推进社会事业发展，增强公共服务能力

加快发展教育事业。推动义务教育均衡发展，建设九年一贯制学校10所，改造农村中小学危房2万平方米。确保白沙湾九年一贯制学校、松山新区曼哈顿小学投入使用，义县“提高普九”顺利通过省评估验收。推进市职教园区工程建设，加快中等职业学校布局调整。

丰富群众文化生活。继续办好古塔庙会、古玩文化节等大型节庆活动。进一步加强文化遗产普查和保护工作。争创省级文化产业示范基地1个。建设乡镇综合文化站20个、农家书屋400个。加快推进体育场馆迁建工程建设。提高竞技体育水平。新建农民健身示范广场30个。全面完成城区25万户数字电视整体平移工程。

加强医疗卫生服务。强化社区卫生服务功能，完善农村三级医疗服务体系，全面提高城乡基本医疗服务水平。整合城市卫生资源，积极推进“一个中心、多个专科”建设。加强公共卫生服务，完善紧急救援中心体系建设。完成传染病院二期、中医院门诊楼、康宁医院整体搬迁工程。进一步落实计划生育家庭奖励扶助政策，全面实施家庭健康促进计划，综合治理人口性别比升高问题，人口出生率和自然增长率分别控制在6.56‰和1.86‰以内。

推进精神文明建设和其它社会事业发展。广泛开展群众性精神文明创建活动，切实把“建设文明锦州、争创文明单位、争做文明市民”活动引向深入。依法管理宗教事务。积极做好省第七届少数民族传统体育运动会前期准备工作。深入开展“五五”普法宣传。巩固双拥成果，落实好各项优抚安置政策。加大社区建设和村民自治工作力度。全力开展好第二次经济普查。切实做好人防、档案、外事、残联、地震等工作，积极发挥铁路、电业、通讯、烟草等中省直单位的作用。

（九）完善各项惠民政策，维护群众切身利益

做好就业再就业工作。继续推进创业带就业工程，不断完善创业孵化基地建设，进一步建立健全就业援助长效机制。切实做好新增劳动力、新失业人员和进城务工人员就业创业工作，实现实名制安置就业5.5万人、普惠制就业培训3.5万人、劳务输出9万人。努力实现零就业家庭动态为零，登记失业困难家庭大学生全部就业。

完善社会保障体系。突出抓好政策完善、保险扩面和基金监管三个重点环节，巩固养老保险扩面成果，推进城镇居民医疗保险工作，切实解决吉祥村为重点的被征地农民保险问题。超额完成省里规定的城镇职工基本养老、医疗、失业、工伤、生育保险指标。全面落实城乡低保各项政策，城乡低保标准在现有基础上分别提高20%和25%以上。贯彻落实《劳动合同法》，着力构建和谐稳定的劳动关系。加大廉租住房和经济适用房建设力度，有效解决城市低收入群体住房困难。认真做好救灾、慈善、助学、济困、助残、法律援助等社会救助工作，推进福利事业健康发展。

维护社会和谐稳定。加强社会治安综合管理，巩固深化公安“三基”工程建设成果，推进公安信息化防控体系建设，严厉打击各类违法犯罪，确保社会平安稳定。加强应急管理体系建设，妥善处置突发公共事件。建设市、县两级“一站式”群众信访接待大厅，健全领导信访接待日和领导包案制度，及时解决群众合理诉求，切实维护群众合法权益。继续做好平抑物价工作，严厉打击价格违法行为。深入实施食品药品安全专项治理和流通领域市场监管，整顿规范市场经济秩序。深入开展安全生产“行政执法年”活动，明确责任主体，加强行政执法，强化煤矿、非煤矿山、危化企业、道路交通和消防安全管理，坚决遏制重特大事故发生。

（十）加强政府自身建设，不断提升行政效能

坚持依法行政。严格执行人大及其常委会的决议、决定，自觉接受人大法律监督、政协民主监督和群众社会监督，切实做好人大代表议案、建议和政协提案办理工作。进一步加大政务公开工作力度。全面落实《锦州市全面推进依法行政五年规划》，不断提高依法行政能力。

强化服务意识。创新服务体制，改进服务方式，进一步强化社会管理和公共服务职能。巩固第四次行政审批制度改革成果，严格落实《服务承诺制》、《首问责任制》、《限时办结制》、《行政处罚自由裁量权暂行规定》等规章制度。切实加强电子政务和政府网站建设，完善政府绩效评价标准和指标体系，全面提高行政效率和服务水平。

切实转变作风。认真落实党风廉政建设责任制，深入开展行政监察、审计监督和纠风专项治理活动，进一步规范各级政府和公务人员的行政行为。继续保持只争朝夕、埋头苦干、锐意进取的精神状态，努力建设一支恪尽职守、清正廉明、勤政为民的公务员队伍。

各位代表，抢抓机遇才能争取主动，把握现在才能赢得未来。让我们在市委的坚强领导下，紧紧依靠全市人民，抢抓机遇，迎难而上，为加速辽西沿海经济区中心城市建设而努力奋斗！
